# Combined improved A* and greedy algorithm for path planning of multi-objective mobile robot

**DOI:** 10.1038/s41598-022-17684-0

**Published:** 2022-08-02

**Authors:** Dan Xiang, Hanxi Lin, Jian Ouyang, Dan Huang

**Affiliations:** 1 School of Automation, Guangdong Polytechnic Normal University, Guangzhou, 510665 Guangdong China; 2 School of Computer Science and Information Engineering, Guangzhou Maritime University, Guangzhou, 510725 Guangdong China; 3 Industrial Training Center, Guangdong Polytechnic Normal University, Guangzhou, 510665 Guangdong China; 4 The School of Mechanical and Automotive Engineering, South China University of Technology, Guangzhou, 510641 Guangdong China

**Keywords:** Energy science and technology, Engineering

## Abstract

With the development of artificial intelligence, path planning of Autonomous Mobile Robot (AMR) has been a research hotspot in recent years. This paper proposes the improved A* algorithm combined with the greedy algorithm for a multi-objective path planning strategy. Firstly, the evaluation function is improved to make the convergence of A* algorithm faster. Secondly, the unnecessary nodes of the A* algorithm are removed, meanwhile only the necessary inflection points are retained for path planning. Thirdly, the improved A* algorithm combined with the greedy algorithm is applied to multi-objective point planning. Finally, path planning is performed for five target nodes in a warehouse environment to compare path lengths, turn angles and other parameters. The simulation results show that the proposed algorithm is smoother and the path length is reduced by about 5%. The results show that the proposed method can reduce a certain path length.

## Introduction

In recent years, warehouse storage^1,2^ has gradually developed in the direction of intelligence and systematization by the "Made in China 2025" strategy^3^ and the rapid development of the logistics industry.

Autonomous Mobile Robot(AMR) which plays an important role in the intelligent process^4^, is widely used in warehouses, hospitals, factories, and transportation industries. Path planning is one of the key technologies of automatic navigation and task scheduling for AMR. Therefore, how to make AMR plan the optimal path while reducing energy consumption and improving the efficiency of the whole storage system is a topic of research for many scholars.

The path planning of AMR is a constrained optimization problem. The algorithms include Genetic algorithm^4^, Probabilistic Roadmap^5^, Rapidly-exploring-random Tree^3,6^, Dijkstra algorithm^7^, A* algorithm^8–10^, Machine learning algorithm^11–13^, Ant Colony algorithm^14^, Particle Swarm Optimization^15^, Artificial potential field algorithm^16,17^ and Breath First Search algorithm^18^, and so on.

Path planning algorithms explore collision-free paths between start point and target point based on map environment information^19^. Huang et al.^20^ introduced the competitive strategy in the standard particle swarm optimization algorithm to find the optimal solution. Li et al. ^21^ designed a multi-objective automated guided vehicles (AGVs) path planning algorithm based on an improved ant colony algorithm. The algorithm can effectively ensure high safety and low energy consumption of AGVs in the logistics and storage environment. Yi et al.^3^ used a random sampling method based on potential function to improve the RRT* algorithm. Also remove the redundant nodes to make the path follow smoothly. Xue et al.^22^ proposed a multi-objective method to solve the multi-objective programming problem.

According to the different characteristics of AMR algorithms, the algorithm can be divided into global path planning and local path planning^23^. In the global planning algorithm, A* algorithm is a heuristic global path planning algorithm and one of the most efficient direct search methods for finding the shortest path in a static environment. Many researchers have improved the A* algorithm, such as bidirectional A* algorithm^24^, A* algorithm based on obstacle information^25^, and so on.

Most researchers have changed the convergence speed of A* algorithm by improving the evaluation function. Wang et al.^10^ proposes a path planning method for improving the A* algorithm by weighting the heuristic function to improve the computational efficiency. Shang et al.^26^ proposed a guideline generated by globally planning to develop heuristic functions and variable-step A* algorithms. Xiong et al.^27^ applied a path processing method based on the A* algorithm to ensure the stability of the vehicle and improve the tracking accuracy by addressing the problems of the A* algorithm not considering vehicle contours and the lack of speed planning. Wang et al.^28^ proposed an improved A* algorithm that introduces turning factors, which solved the shortest path problem of multiple AGVs. Zheng et al.^29^ added the angle evaluation cost function to the cost function of A* algorithm and used the feature of jump point search to improve the search speed.

The traditional A* algorithm has the problem of redundant nodes. Zhang et al.^30^ introduced a key point selection strategy for secondary planning of the path, which deleted redundant turning nodes and invalid nodes. Guruji et al.^31^ proposed an improvement of the A* algorithm to reduce the processing time by determining the value of the heuristic function before the collision phase. Quan et al.^32^ studied the A* algorithm for grid path planning at different obstacle scales. Meanwhile, an improved A* algorithm is introduced to optimize the key points and simplify the paths to key points. Song et al.^33^ proposed an improved algorithm to solve the problem that traditional A* algorithm were constrained by map resolution.

In multi-objective planning, Melo et al.^34^ considered the presence of many objects in the environment to minimize the time taken by all robots to reach the target location and to reduce the distance traveled by the robots. The problem is modeled as a graph problem in which a path is decomposed into segments and the robots dynamically choose the best path to execute greedily in a certain time. Faridi et al.^35^ proposed an evolutionary solution to the multi-intelligent, multi-objective navigation problem in an unknown dynamic environment. Combination of the improved artificial swarm and evolutionary planning is used to smooth remove the resulting intermediate feasible paths. Ayomoh et al.^36^ used the principle of the shortest distance between the robot and the target to calculate the order of access to the target point. This approach minimizes the path time of the mobile robot. Yang et al.^37^ proposed an adaptive multi-objective genetic differential evolution algorithm for multi-objective scheduling to achieve multi-objective scheduling optimization of AGV systems under multi-constraint conditions.

Aiming at the problem that the A* algorithms were rarely used in multi-objective planning. Wang et al.^38^ used a start-to-target cost function to rank target sequences and applied the improved A* algorithm to multi-objective point planning. Yue et al.^39^ decomposed the AMR target region into multiple sub-target points and used an improved simulated annealing algorithm for multi-objective point path planning.

## Environment modeling

Environment modeling is an essential step in the path planning of the A* algorithm and the basis for the subsequent steps. For A* algorithm, environment modeling is gridding the map. Therefore, the size of the grid of the rasterized map will affect the planning quality of the mobile robot^8^.

### Grid modeling

The principle of the grid method is to split the entire space into grids. Each grid represents the environmental information of its location. The modeling steps are as follows^8^:

(i) Splitting the whole space into grids of the same size;
(ii) Corresponding each grid to the location area in the actual environment space;
(iii) Set the status of each grid to distinguish whether the corresponding area can pass.

**Figure 1:**
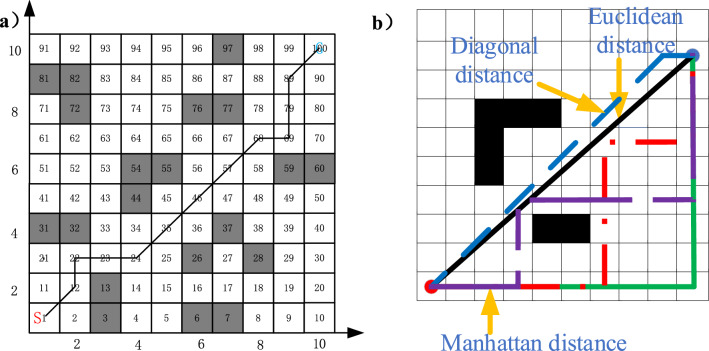
(**a**) Schematic diagram of grid method (**b**) Paths generated by different distance functions.

As shown in Fig. 1, the obstacle's position is marked as the black part, which means AMR cannot pass, and the white area is the feasible area. The start point is represented by *S*, and end point is represented *G*. There are two ways to mark the grid position.

(i) Coordinate method: The horizontal and vertical axes are established with the two edges of the grid map. The horizontal direction is the *x*-axis and the vertical direction is the *y*-axis. Then the position of the grid can be marked by the coordinates (*x, y*).
(ii) Sequential method: Each grid is marked incrementally in a horizontal and vertical pattern, with each grid having its number.

The two marking modes can be transformed into each other and their correspondence is

1 $$ N = x + n \times y $$

2 $$ \left\{ {\begin{array}{*{20}c} {x_{i} = [(N_{i} - 1)/n] + 1} \\ {y_{i} = [(N_{i} - 1)/n] + 1} \\ \end{array} } \right. $$

where ${x}_{i}$ and ${y}_{i}$ are the horizontal and vertical coordinates of the grid map. *N* represents the grid number under the sequence method, *n* is the grid splitting fineness. [] is the rounding down operation.

The feasible route is shown in Fig. 1a. According to the coordinate method M1 and the sequence method M2, the grid number of the route can be expressed as:

M1 = [(1,1), (2,2), (2,3), (3,3), (4,3), (5,4), (6,5), (7,6), (8,7), (9,7), (9,8), (9,9), (10,10)].

M2 = [1, 12, 22, 23, 24, 35, 46, 57, 68, 69, 79, 89, 100].

In this paper, the coordinate method is used to represent the motion environment of AMR. The advantage of grid modeling is that it greatly reduces the difficulty of abstracting complex environments. The disadvantage is that grid modeling is difficult to determine the size of the grid division. If the grid is too small, it will increase the complexity of the subsequent search algorithm and take up a lot of memory. If the grid is too large, it will not correctly represent the real environment, and there is a possibility of collision in the subsequent planning of the AMR. Therefore, when using the grid method to model the path planning environment, it is necessary to weigh the actual environment and the requirements of the search algorithm to choose the division standard^40^.

### Path planning objective function

AMR needs to return to the charging station to recharge when the battery power is insufficient. At this time, two issues need to be considered: first, whether the remaining power can support the AMR to reach the recharge point; second, the current task performed depends on whether the AMR can be abandoned and what the trade-off is for urgent tasks.

Therefore, the remaining power of AMR must consider the most complicated situation. When the path is planned for the furthest case, the AMR should be able to return to the charging point while avoiding the obstacles at the farthest target point. At this time, the total distance of the A* planning objective function is *L*_*path*_. The formula *L*_*path*_ is as follows:

3 $$ L_{path} = \sum\limits_{i = 1}^{m} {\sqrt {(x_{i + 1} - x_{i} )^{2} + (y_{i + 1} - y_{i} )^{2} } } $$

where $(x_{i} ,y_{i} )$ is the coordinates of the *i*-th target point in the path of the AMR. At the same time, the safe operation of the path planned needs the AMR to acquire an angle which smaller than the maximum turning angle:

4 $$ \left\{ \begin{gathered} \theta \left( {n_{i - 1} ,n_{i} ,n_{i + 1} } \right) \le \theta_{\max } \hfill \\ 180^{ \circ } { - }\theta (n_{i - 1} ,n_{i} ,n_{i + 1} ) < 90^{ \circ } \hfill \\ \end{gathered} \right. $$

where, *n*_*i-*1_, *n*_*i*_, and *n*_*i*+1_ are the previous node, the current node, and the next node, respectively. ${\theta }_{max}$ represents the maximum safe turning angle of the AMR to avoid overturning due to running inertia, which can be set according to the actual situation.

## Traditional A* algorithm

The traditional A* algorithm is a heuristic search algorithm that can realize path planning in a global static environment. The evaluation function of the A* algorithm is: *F*(*n*) = *G*(*n*) + *H*(*n*).

### Valuation function of path algorithm

*G*(*n*) and *H*(*n*) in the evaluation function are a kind of mutual restriction relationship. *H*(*n*) belongs to the heuristic function and is an important part of the evaluation function *F*(*n*). The choice of *F*(*n*) will directly affect the quality of the A* algorithm.

In the A* algorithm, if the estimated cost *H*(*n*) is too small, the actual cost *G*(*n*) is too large. The role of *G*(n) in the evaluation function *F*(n) takes the major part, the algorithm will be simplified to Dijkstra's algorithm and the computational effort will increase. If the actual cost *G*(*n*) is too small and the estimated cost *H*(*n*) is large, the algorithm will be simplified to the BFS algorithm and the obtained path will not be guaranteed to be optimal.

Manhattan distance ${c}_{1}$, Euclidean distance ${c}_{2}$ and diagonal distance ${c}_{3}$ can all be used in the A* algorithm to calculate the cost function *F*(n). The manhattan distance ${c}_{1}$, the euclidean distance ${c}_{2}$, and the diagonal distance ${c}_{3}$ are shown in formula (5):

5 $$ \left\{ \begin{gathered} c_{1} = \left| {x_{g} - x_{n} } \right| + \left| {y_{g} - y_{n} } \right| \hfill \\ c_{2} = \sqrt {{(}x_{g} - x_{n} {)}^{2} + {(}y_{g} - y_{n} {)}^{2} } \hfill \\ c_{3} = 1.4 \times \min \left( {\left| {x_{g} - x_{n} } \right|,\left| {y_{g} - y_{n} } \right|} \right) + \left( {\left( {\left| {x_{g} - x_{n} } \right| + \left| {y_{g} - y_{n} } \right|} \right) - 2 \times \min \left( {\left| {x_{g} - x_{n} } \right|,\left| {y_{g} - y_{n} } \right|} \right)} \right) \hfill \\ \end{gathered} \right. $$

Here, the start node coordinate is $\left( {x_{n} ,y_{n} } \right)$ and the target coordinate is $(x_{g} ,y_{g} )$.

As shown in Fig. 1b, the difference between the three can be clearly seen. The number of turns for Euclidean distance is 0, and the distance is also the shortest. Therefore, Euclidean distance is selected in this paper.

## Based on the improved A* algorithm path planning

### Evaluation function design based on obstacle information

The A* algorithm is one of the effective algorithms for searching the shortest path in a static environment. The traditional evaluation function is *F*(*n*) = *G*(*n*) + *H*(*n*).

Therefore, the evaluation function is improved as follows:

6 $$ F\left( n \right) = G\left( n \right) + H\left( n \right) + o\left( n \right) $$

where,

7 $$ \left\{ \begin{gathered} o(n) = - \alpha C(n) + \beta {\rm I}(n) \hfill \\ C(n) = \frac{1}{50}\frac{R - r}{R}H(n) \hfill \\ I\left( n \right) = \frac{1}{20}\sum\limits_{i = 1}^{M} {\frac{1}{{\sqrt {\left( {x_{i} - x_{s} } \right)^{2} + \left( {y_{i} - y_{s} } \right)^{2} } }}} \hfill \\ \alpha + \beta = 1 \hfill \\ \end{gathered} \right. $$

where *C*(*n*) is the information weight biased towards the target point and is the information value of the next node around the current node to the target point. The closer the next node is to the target point, the smaller the total *H*(*n*). *r* is the distance from the adjacent coordinate point of the current point to the target point. *R* is the distance from the start point to the target point.

*I*(*n*) is called the prediction function based on obstacle information. Where *M* is the number of obstacles on the path from the current node to the target point. *I*(*n*) represents the obstacle information in the expansion direction of the target point. The closer the current node is to the obstacle, the greater cost of moving along the current direction. This mechanism has a positive guiding effect on the path search and can guide the path to avoid near obstacles early. The more obstacles are distributed along the current expansion direction line, the more expensive it is for the node to expand along that direction. This mechanism also has a positive effect on path navigation, guiding the route to avoid density areas of obstacles at an early stage. These two mechanisms can reduce the number of extended nodes, thereby improving the search efficiency of the algorithm.

$\alpha$ and $\beta$ are the weight values, and the value of $\beta$ is the ratio of the line between the start points and target points about the contact with the obstacle. When $\beta { = 0}$, there is no object on the path from the current node to the target point. So, the heuristic function *H*(*n*) weight can be increased appropriately to reduce the search range and improve the search efficiency. When $\beta { = 1}$, all the obstacles exist on the path from the current node to the target point. So, the weight of the heuristic function *H*(*n*) is not changed to increase the search range and avoid falling into the local optimum.

### Node optimization

The traditional A* algorithm searches in 8 directions around the current node. The advantage of omnidirectional expansion is that the algorithm can adapt to more complex obstacle environments. But the disadvantage is that the number of expansion directions inevitably reduces the efficiency of the algorithm's search. In most cases, the obstacles are not so complex that omnidirectional node expansion is not required to complete the route planning.

In this paper, an adaptive method of node search direction is proposed. First, find the angle $\theta$ formed by the line joining the current node $(x_{0} ,y_{0} )$ and the target point $(x_{g} ,y_{g} )$ with the direction of the x-axis. $\theta$ is mainly judged based on the angle between the line at the start point, target point, and x-axis. $\theta$ can be obtained from formula (8).

8 $$ \theta {\text{ = arctan}}\frac{{y_{g} - y_{0} }}{{x_{g} - x_{0} }} $$

where,

9 $$ \theta = \left\{ {\begin{array}{*{20}l} {90^{ \circ } } \hfill & {y_{g} > y_{0} \cap x_{g} = x_{0} } \hfill \\ {270^{ \circ } } \hfill & {y_{g} < y_{0} \cap x_{g} = x_{0} } \hfill \\ {{\text{arctan}}\frac{{y_{g} - y_{0} }}{{x_{g} - x_{0} }}} \hfill & {y_{g} > y_{0} \cup x_{g} \ne x_{0} } \hfill \\ {180^{ \circ } + {\text{arctan}}\frac{{y_{g} - y_{0} }}{{x_{g} - x_{0} }}} \hfill & {y_{g} < y_{0} \cup x_{g} \ne x_{0} } \hfill \\ \end{array} } \right. $$

Second, the current node is expanded into a 90° sector with a radius $r = \sqrt 5$. If there are no obstacles in the identification area, the eight directional search sub-nodes are changed to 3 search directions, and the following steps are performed to further reduce the search time. The specific rules are shown in Table 1.

**Table 1:** Three-direction rule table.

$\theta$	Keep 3 directions	Abandon direction
[337. 5°, 360°) ∪ [0°, 22. 5°)	315 T, 000 T, 045 T	090 T, 135 T, 180 T, 225 T, 270 T
[22. 5°, 67. 5°)	000 T, 045 T, 090 T	135 T, 180 T, 225 T, 270 T, 315 T
[67. 5°, 112. 5°)	045 T, 090 T, 135 T	000 T, 180 T, 225 T, 270 T, 315 T
[112. 5°, 157. 5°)	090 T, 135 T, 180 T	045 T, 225 T, 270 T, 315 T, 000 T
[157. 5°, 202. 5°)	135 T, 180 T, 225 T	000 T, 045 T, 090 T, 270 T, 315 T
[202. 5°, 247. 5°)	180 T, 225 T, 270 T	000 T, 045 T, 090 T, 135 T, 315 T
[247. 5°, 292. 5°)	225 T, 270 T, 315 T	045 T, 090 T, 135 T, 180 T, 000 T
[292. 5°, 337. 5°)	270 T, 315 T, 000 T	045 T, 090 T, 135 T, 180 T, 225 T

Otherwise, when there are less than four obstacles in the recognition area, five nodes are used to search for directions. The specific rules are shown in Table 2. In other cases, it is restored to 8 search nodes.

**Table 2:** Five-direction rule table.

$\theta$	Keep 5 directions	Abandon direction
[337. 5°, 360°) ∪ [0°, 22. 5°)	000 T, 045 T, 090 T, 270 T, 315 T	135 T, 180 T, 225 T
[22. 5°, 67. 5°)	000 T, 045 T, 090 T, 135 T, 315 T	180 T, 225 T, 270 T
[67. 5°, 112. 5°)	000 T, 045 T, 090 T, 135 T, 180 T	225 T, 270 T, 315 T
[112. 5°, 157. 5°)	045 T, 090 T, 135 T, 180 T, 225 T	270 T, 315 T, 000 T
[157. 5°, 202. 5°)	090 T, 135 T, 180 T, 225 T, 270 T	000 T, 045 T, 315 T
[202. 5°, 247. 5°)	135 T, 180 T, 225 T, 270 T, 315 T	000 T, 045 T, 090 T
[247. 5°, 292. 5°)	180 T, 225 T, 270 T, 315 T, 000 T	045 T, 090 T, 135 T
[292. 5°, 337. 5°)	225 T, 270 T, 315 T, 000 T, 045 T	090 T, 135 T, 180 T

### Route optimization

**Figure 2:**
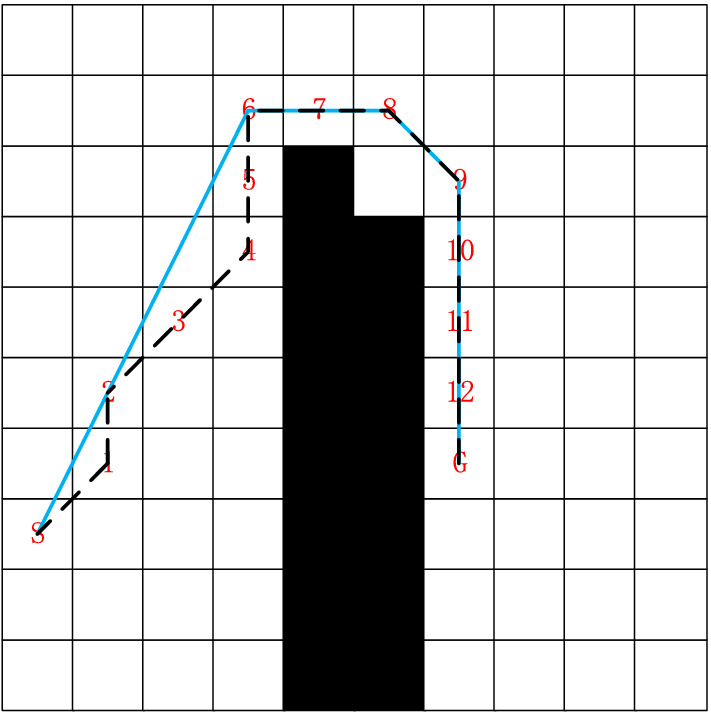
Schematic diagram of path smoothing optimization.

Traditional A* algorithm path planning consists of continuous grid centroid connections, with many redundant nodes, the path turns, and unsmooth paths. The path smoothing optimization algorithm is designed based on the Floyd algorithm idea to address these problems. The principle of path smoothing optimization is shown in Fig. 2. As an example, the path planned by the traditional A* algorithm is (S, 1, 2, 3, 4, 5, 6, 7, 8, 9, 10, 11, 12, 13, G), and there are many redundant nodes. The Floyd idea is used to retain the key nodes of the path and optimize the path by removing the redundant inflection points. The improved path is relatively smooth, while reducing the path length and inflection points. The steps of path smoothing optimization are as follows:

Step 1: Output the final path generated by the improved A* algorithm before optimization.
Step 2: Iterate through all nodes and determine the inflection point. Judge whether $k_{1}$ and $k_{2}$ are equal by formulas (10) and (11). When $k_{1} \ne k_{2}$, $(x_{i} ,y_{i} )$ is the inflection point. The original path keeps only the start point *S* and the target point *G* and the inflection point (1,2,4,6,8,9), and uses the new coordinates $(x_{n} ,y_{n} )$ to distinguish the inflection nodes.
  10 $$ k_{1} = \frac{{y_{i} - y_{i - 1} }}{{x_{i} - x_{i - 1} }} $$
  11 $$ k_{2} = \frac{{y_{i + 1} - y_{i} }}{{x_{i + 1} - x_{i} }} $$
  where $(x_{i - 1} ,y_{i - 1} )$, $(x_{i} ,y_{i} )$, and $(x_{i + 1} ,y_{i + 1} )$ are the three adjacent nodes of the final path generated by the improved A* algorithm before output optimization.
Step 3: Iterate through the start point and the inflection points. Set the three adjacent inflection points $(x_{n - 1} ,y_{n - 1} )$ , $(x_{n} ,y_{n} )$ , and $(x_{n + 1} ,y_{n + 1} )$ as a group, and determine the relationship between the path d between the first and third inflection points and the path *D* between the three inflection points. *D* and *d* are shown in formulas (12) and (13). If *d* <  = *D* and *d* does not pass through obstacles, the first and third nodes are reserved; otherwise, three nodes are reserved.
  12 $$ D = \sqrt {(x_{n + 1} - x_{n - 1} )^{2} + (y_{n + 1} - y_{n - 1} )^{2} } $$
  13 $$ d = \sqrt {(x_{n - 1} - x_{n} )^{2} + (y_{n - 1} - y_{n} )^{2} } + \sqrt {(x_{n + 1} - x_{n} )^{2} + (y_{n + 1} - y_{n} )} $$
  where $(x_{n - 1} ,y_{n - 1} )$, $(x_{n} ,y_{n} )$, and $(x_{n + 1} ,y_{n + 1} )$ are three adjacent turning nodes.
Step 4: Extract the remaining nodes, output the optimized path. The algorithm ends.

In path planning, when AMR encounters an obstacle, it needs to perform a turning action. The turning angle of AMR is determined by using the formula (14).

14 $$ angle = \arccos \left( {\frac{{(x_{n - 1} - x_{n} )(x_{n + 1} - x_{n} ) + (y_{n - 1} - y_{n} )(y_{{{\text{n + }}1}} - y_{n} )}}{{\sqrt {(x_{n - 1} - x_{n} )^{2} + (y_{n - 1} - y_{n} )^{2} } \sqrt {(x_{n + 1} - x_{n} )^{2} + (y_{n + 1} - y_{n} )^{2} } }}} \right). $$

## Single objective verification

To verify the effectiveness and feasibility of the improved A* algorithm, the simulation software platform is PyCharm, and the hardware platform is Intel(R) Core(TM) i7-7700 CPU @ 3.60 GHz, 8 GB memory, 64-bit operating system, Windows 10 computer.

There are three main indicators for evaluating the quality of a planned path: path length, smoothness, and planning time. The path length is the main indicator and the fundamental problem of optimizing the path. The shorter the path length, the better the algorithm performance. The degree of smoothness affects the speed and safety of the AMR in practical applications. The degree of smoothness is reflected by comparing the total turning angle of the path planning. The planning time is whether the system can quickly make a response strategy when it affects the actual application.

### Three different grid maps

The traditional A* algorithm and the improved A* algorithm are verified on maps of 20 × 20, 30 × 30, and 50 × 50, respectively.

### Simulate on three different grid maps

**Figure 3:**
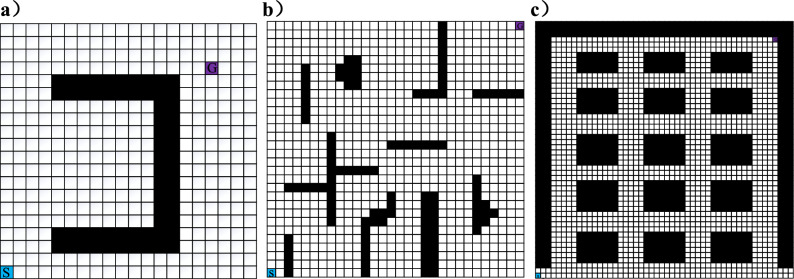
Three different grid maps. (**a**) Map 1 (**b**) Map 2 (**c**) Map 3.

In this paper, we will perform 10 simulations on three different grid maps to compare the performance differences between the A* algorithm and the improved A* algorithm. This requires determining the start point coordinates and the target point coordinates. In Fig. 3, the coordinates of the start point of grid map 1, 2 and 3 are all (1, 1) and are represented by the blue area *S*. The coordinates of the target points are (17, 17), (30, 30) and (47, 47), respectively. The target points are represented by the purple area *G*.

**Figure 4:**
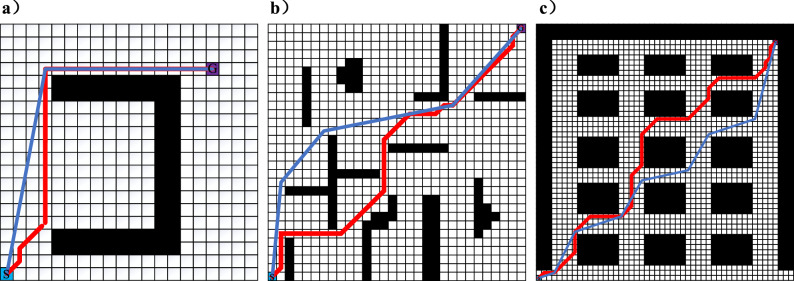
Comparison of the paths generated. (**a**) Map 1 (**b**) Map 2 (**c**) Map 3.

The algorithms were simulated 10 times on these maps to obtain the data in Table 3 and the path diagram in Fig. 4. In Fig. 4, the difference between the A* algorithm and the improved A* algorithm in planning out the routes in three different types of grid maps can be clearly seen. The red route and the blue route are produced by the traditional A* algorithm and the improved A* algorithm, respectively.

**Table 3:** Comparison of three kinds of grid map experiment simulation data.

Map type	Path parameter	Traditional A* algorithm	Improved A* algorithm	Reduction ratio
Map1 (20 × 20)	Planning time/s	0.830	0.742	10.602%
	Number of nodes	202	182	9.900%
	run time/s	60.486	58.558	3.188%
	Number of inflection points	4	1	75%
	Total turning angle	405	100.620	75.156%
	Total distance length	30.243	29.279	3.188%
Map 2 (30 × 30)	Planning time /s	2.055	1.935	5.839%
	Number of nodes	365	401	− 9.863%
	run time /s	94.912	90.732	4.404%
	Number of inflection points	11	2	81.818%
	Total turning angle	1440	429.448	70.177%
	Total distance length	47.456	45.366	4.404%
Map 3 (50 × 50)	Planning time /s	6.548	6.505	0.657%
	Number of nodes	853	796	6.682%
	run time /s	77.355	71.948	6.990%
	Number of inflection points	18	8	55.556%
	Total turning angle	2700	934.270	65.397%
	Total distance length	77.355	71.948	6.989%

From Fig. 4a–c, it can be seen that the total turning angle, the number of turning points, and the total distance length of the improved A* algorithm are significantly reduced compared with the traditional A* algorithm. The improved path has no redundant turning points and is relatively smooth. The results of the runs on Fig. 4a show that the improved A* algorithm does not fall into local optima in the U-shaped obstacle environment, thus generating suboptimal paths. The planning time and the number of planning nodes have been reduced by about 10%. The total distance length has been reduced by 3.188%. The running results of Fig. 4b show that the improved A* algorithm can quickly plan the path, the planning time is reduced by 5.839%, the total distance length is reduced by 4.404%, and the turning angle is reduced by 75.156%. The results of running on Fig. 4c show that the planning time of the improved A* algorithm is similar to that of the conventional A* algorithm for running. But the turning angle is reduced by 65.4% and the total distance is reduced by 6.990%.

In order to verify the performance difference between the improved A* algorithm and other algorithms. In the environment of Fig. 3c, Bestfirst, BFS, RRT and Bidirectional-A* algorithms are used to compare with the improved A* algorithm. The experimental data are shown in Table 4. It clearly shows that the improved A* algorithm performs better in all aspects. Except the number of nodes is larger than that of the Bidirectional-A* algorithm. The path length and turning angle of the improved A* algorithm is significantly shorter.

**Table 4:** Comparison of different algorithm experiments.

Map type	Path parameter	Dijistar	RRT	BFS	Bidirectional A*	Improved A* algorithm	Reduction ratio
Map 3 (50 × 50)	Planning time/s	6.925	10.183	10.897	6.600	6.505	1.439% ~ 40.305%
	Number of nodes	1432	848	1432	664	796	− 19.880% ~ 44.413%
	Run time/s	154.710	178.322	154.710	156.366	143.896	6.990% ~ 19.306%
	Number of turning angles	17	89.3 (exclude)	21	18	8	52.941% ~ 61.905%
	Total turning angle	2295	13,247.17 (exclude)	2700	2340	934.27	59.291% ~ 65.397%
	Total distance /m	77.355	89.161	77.355	78.183	71.948	6.990% ~ 19.306%

Among them, the RRT algorithm generates paths by randomly sampling points, and its paths are not unique. In the environment of Fig. 3c, the RRT algorithm is run 10 times. The average path time is 10.183 s and the average path length is 89.161 m. Compared with the RRT algorithm, the improved A* algorithm reduces the average planning time and the average path length by 36.119% and 19.306%.

In summary, compared with other algorithms, the improved A* algorithm has a small reduction in single-objective planning time. The planned path length and turning angle are significantly reduced. Smoothness is significantly improved.

## Multi-objective verification

The traditional A* algorithm is only suitable for searching a single target point and has the disadvantages of low efficiency and poor compatibility in performing multiple objective point searches. This is difficult to meet the application of AMR in daily life planning the optimal path of multiple objective points. Therefore, an improved A* algorithm combined with the idea of greedy algorithm is proposed in this paper. The A* algorithm is applied to multi-objective search and improves the planning efficiency of multi-objective nodes.

### Improved A* algorithm combined with the greedy algorithm

During the algorithm's execution, *n*-*m*(*m* <= *n*/*2*) intermediate target points are randomly selected and all permutations. The *F*(*n*) function of the improved A* algorithm is used to calculate the node arrangement sequence solution with the shortest overall planning time. Then, the remaining *m* target nodes are inserted into the previous optimal path one by one through the mechanism of the greedy algorithm, and the planning of multiple objective points is achieved at once. Since circular paths are not allowed in this paper, a node can only appear at most once in a path.

In the multi-objective optimal path problem, find all Pareto optimal solution paths between $Start$ and $Target$ as follows: $Start$ is the start point and $Target$ is the final point of AMR. The path of the AMR can be represented as a set consisting of the starting point, the *n* target points passed in between, and the final point, which can be represented as: $Path = \{ Start,X_{1} ,X_{2} ,...,X_{n} ,Target\}$ . Where, the set $P = \{ X_{1} ,X_{2} ,...,X_{n} \}$ is the path optimization objective between the start point and the final point; $X_{i} (i = 1,2,...,n)$ is the path point between the start point and the final point, which is the target location point of the AMR to complete the task, and the coordinates are not in the position of obstacles, and the coordinates of each $X_{i}$ are $(x_{i} ,y_{i} )$ .

The total path distance of multiple objective points is $Ls_{path}$. The calculation formula of $Ls_{path}$ is as follows:

15 $$ Ls_{path} = \min \left( {\sum\limits_{j = 0}^{n - 1} {\sum\limits_{i = 1}^{s} {\sqrt {(x_{i + 1} - x_{i} )^{2} + (y_{i + 1} - y_{i} )^{2} } } } } \right) $$

The specific steps of the improved multi-A* algorithm are summarized as follows:

Step 1: Randomly select *n-m* target points from *m* intermediate target points for full permutation, and add the start point coordinates at the beginning of each arrangement and the final point coordinates at the end. And the sequence of target points is fed into the improved A* algorithm to solve the path sequence with the shortest planning time from all the sequences.
Step 2: Choose a target point arbitrarily from the remaining *m* target points, insert it into the two adjacent target points of the optimal path sequence obtained by Step1, and use the improved A* algorithm to solve for the optimal path in the current case among the *n*-*m + *1 possible path sequences.
Step 3: Select an arbitrary target point from the remaining *m-*1 target points, insert it into the two target points of the optimal path sequence obtained by Step2, and use the improved A* algorithm to solve for the optimal path in the current case among the *n-m + *2 possible path sequences.
Step 4: By analogy, until all *m* target points are selected, the path sequence obtained is optimal.
Step 5: Determine whether to traverse all target nodes. If yes, end the operation. Otherwise, repeat the above steps.

Take the current node path $Path_{4} = \left\{ {SP_{1} P_{4} P_{3} P_{2} G} \right\}$, insert two nodes $P_{5}$ and $P_{6}$, respectively. The specific expressions are as follows:

$$ \begin{array}{*{20}c} {Path_{5} = min\{ SP_{5} P_{1} P_{4} P_{3} P_{2} G,SP_{1} P_{5} P_{4} P_{3} P_{2} G,SP_{1} P_{4} P_{5} P_{3} P_{2} G,SP_{1} P_{4} P_{3} P_{5} P_{2} G{,}} \\ {SP_{1} P_{4} P_{3} P_{2} P_{5} G\} = \{ SP_{1} P_{5} P_{4} P_{3} P_{2} G\} } \\ \end{array} $$

$$ \begin{array}{*{20}c} {Path_{6} = min\{ SP_{6} P_{1} P_{5} P_{4} P_{3} P_{2} G,SP_{1} P_{6} P_{5} P_{4} P_{3} P_{2} G,P_{1} P_{5} P_{6} P_{4} P_{3} P_{2} G,SP_{1} P_{5} P_{4} P_{6} P_{3} P_{2} G,} \\ {SP_{1} P_{5} P_{4} P_{3} P_{6} P_{2} G,SP_{1} P_{5} P_{4} P_{3} P_{2} P_{6} G\} = \{ SP_{1} P_{5} P_{4} P_{3} P_{6} P_{2} G\} } \\ \end{array} $$

### Verify the feasibility of the greedy algorithm

This paper verifies the greedy algorithm's feasibility using the traditional A* algorithm on a simple grid map of 50 × 30. The start and final points of the multi-objective planning are (1,1) and (50,30), respectively. There are 6, 7, 8, and 9 intermediate target nodes between the start and final points.

The intermediate target nodes of the above four cases are fed into the traditional A* algorithm, and *n-m* intermediate target nodes are randomly selected for full permutation to plan the minimum time target sequence. In this paper, ten simulation experiments are conducted by using the greedy algorithm to insert the *m* intermediate target nodes into the current optimal sequence of *n-m* intermediate target nodes one by one and derive the average planning path time.

Table 5 shows that the average planning time of the algorithm gradually decreases with the insertion of more intermediate target nodes and reaches the minimum average planning time at the insertion of about *n*/2 intermediate target points.

**Table 5:** Average time table of inserted node planning path.

	Total number of nodes		
Number of inserted nodes	8	9	10
1	4.674	26.323	126.589
2	2.268	5.356	27.453
3	1.837	2.892	6.613
4	1.898	2.450	3.664
5	2.064	2.377	3.220
6	2.063	2.575	3.302
7	–	2.504	3.233
8	–	–	3.137

Using the greedy algorithm to insert the remaining intermediate target nodes one by one when approaching *n*/2 nodes, the greedy algorithm's efficiency is the highest. After that, there is a slight rebound phenomenon. The reason is that the more nodes inserted, the more times the program needs to be run. Therefore, we choose to insert *n*/2 nodes in the follow-up of this experiment.

In summary, it is verifies that the greedy algorithm is feasible in path planning.

### Greedy algorithm combined with improved A* algorithm

**Figure 5:**
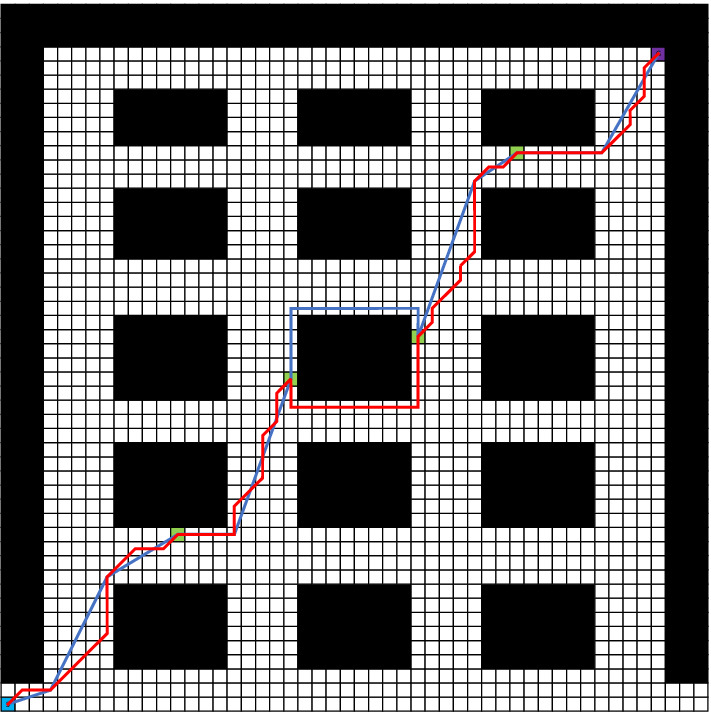
Multi-objective path planning.

The improved A* algorithm is fused with the greedy algorithm so that the improved A* algorithm can be applied in multi-objective path planning. The start point is (1,1), and the final point is (47,47). The coordinates of the intermediate target nodes are (13,13), (21,24), (30,27) and (37,40). The simulation of multiple intermediate target nodes is performed ten times in Fig. 3c. The specific run results are shown in Fig. 5.

In Fig. 5, the blue route is the path generated by the improved algorithm, while the red route is the route generated by the idea of applying the A* algorithm to multiple objective in the literature^38^. It can be seen intuitively that the length of the blue route is shorter than that of the red route. The specific data are shown in Table 6. Although the average planning operation time of the improved algorithm is increased by 14.694% compared with the method in literature^38^, the average path planning length and total turning angle are reduced by 4.931% and 62.641%, respectively. The significant decrease in the total turning angle improves the smoothness when the AMR moves, which is beneficial to the safety of the AMR and the cargo.

**Table 6:** Comparison of multi-objective experiments.

Parameter	Improved algorithm	Literature^38^	Reduction ratio
Average planning time	1.686	1.470	− 14.694%
Average planned path length	79.015	83.113	4.931%
Total turning angle	1311.276	3510	62.641%
Number of nodes	207	250	17.2%
Average running time	158.030	166.226	4.931%

This method is suitable for AMR to process orders at multiple objective points. AMR can perfectly respond to emergency events or task changes. On the one hand, in a sudden emergency, the method can quickly insert coordinate task points into the task sequence to get a mobile target sequence. AMR will complete the current urgent task. For example, when the AMR is low on power and needs to be charged, the charging node is inserted. On the other hand, when dealing with newly added task target points, the task coordinate points can be quickly inserted into the obtained mobile sequence of AMR to get a new shortest mobile target sequence.

### Algorithm complexity analysis

The traditional A* algorithm has a worst-case scenario in which the actual cost function in the evaluation function takes the major part. In this case, the algorithm will be simplified to the Dijkstra algorithm, and the amount of calculation will increase. The algorithm needs to visit all unmarked nodes when selecting the shortest path node, which is inefficient. The running time of the entire algorithm for a single target point is $o\left( {s^{2} } \right)$, where $s$ is the number of nodes. The time complexity of the literature^38^ is $o\left( {s^{2} } \right)$. Literature^38^ occupies an obvious advantage in planning speed, but the method only considers the direction of the start point toward the final point and cannot be used in an omnidirectional environment. When applied in the case that all target points are not in the same direction, the solved path is not necessarily the optimal solution with the shortest path length.

Compared to the globally optimal path planning obtained by traversing all intermediate target nodes using the exhaustive method, the time complexity of the entire algorithm for traversing all intermediate target nodes is $o((n!)s^{2} )$, in the presence of a large number of intermediate target points. The method will spend a lot of time optimally ranking the path plan's nodes. And the running time of the improved A* algorithm proposed in this paper is mainly determined by n-m target points, so the time complexity of the entire algorithm is about $o\left( {\left( {{(}n - m{)! + }\sum\nolimits_{i = 1}^{m} {{(}n - m - i{)}} } \right)s^{2} } \right) \approx o\left( {\left( {{(}n - m{)!}} \right)s^{2} } \right)$. Therefore, the time complexity of the improved algorithm proposed in this paper depends on *n-m* target points for full permutation, and then the improved A* algorithm is used to find the path node arrangement with the shortest time.

## Conclusion

In order to improve the efficiency of AMR path planning under multi-objective conditions, this paper proposes an improved A* algorithm combined with greedy algorithm idea to realize the A* algorithm for multiple objective point planning. The combined algorithm allows path planning for multiple objective points. The efficiency of path planning is improved by improving the evaluation function. The nodes are also optimized to make up for the shortcomings of the traditional A* algorithm with poor timeliness. At the same time, the planned path length and turning angle are significantly shortened. Through comparative analysis, the effectiveness of the algorithm is verified. In the future, the path planning problem of mobile robots under multi-tasking conditions in dynamic environments and smulated annealing will be studied and the algorithm should be applied in AMR.

## Data Availability

The datasets used and analysed during the current study available from the corresponding author on reasonable request.
